# Consideration of Surrogate Endpoints for Overall Survival Associated With First-Line Immunotherapy in Extensive-Stage Small Cell Lung Cancer

**DOI:** 10.3389/fonc.2021.696010

**Published:** 2021-07-14

**Authors:** Shuang Zhang, Shuang Li, Yanan Cui, Peiyan Zhao, Xiaodan Sun, Ying Cheng

**Affiliations:** ^1^ Department of Thoracic Oncology, Jilin Cancer Hospital, Changchun, China; ^2^ Big Data Center of Clinical, Jilin Cancer Hospital, Changchun, China; ^3^ Postdoctoral Research Workstation, Jilin Cancer Hospital, Changchun, China

**Keywords:** small-cell lung cancer, surrogate endpoints, survival, immunotherapy, restricted mean survival time (RMST)

## Abstract

**Background:**

The combination of immune checkpoint inhibitors (ICIs) and chemotherapy is known to improve overall survival (OS) in patients with extensive-stage small cell lung cancer (ES-SCLC). ICIs have different response patterns and survival kinetics characteristics from those of the traditional chemotherapy. In first-line treatment for ES-SCLC, there is an urgent need for surrogate endpoints for the early and accurate prediction of OS. This study aimed to assess progression-free survival (PFS), milestone OS rate, milestone restricted mean survival time (RMST), overall response rate (ORR), and disease control rate (DCR) as proposed surrogate endpoints for OS in ES-SCLC for first-line immunotherapy trials.

**Methods:**

Between January 1, 2013, and December 2020, published articles on randomized clinical trials of ICIs plus chemotherapy in patients with ES-SCLC as first-line therapy were searched in PubMed. Abstracts from the ESMO, ASCO, and WCLC, reported from 2018 onwards, were also searched. A weighted regression analysis based on the weighted least squares method was performed on log-transformed estimates of treatment effect, and the determination coefficient (R^2^) was calculated to evaluate the association between treatment effect on the surrogate endpoint and OS.

**Results:**

Seven trials, representing 3,009 patients, were included to make up a total of 16 analyzed arms. The ratio of the 12-month OS milestone rate (r = −0.790, P = 0.011, R^2^ = 0.717) and 12-month OS milestone RMST (r = 0.798, P = 0.010, R^2^ = 0.702) was strongly correlated with the hazard ratio (HR) for OS. The strongest association was observed between the ratio of the 24-month OS milestone RMST and the HR for OS (r = 0.922, P = 0.001, R^2^ = 0.825). No associations were observed between the HR for OS and PFS and the RR for ORR and DCR.

**Conclusions:**

The results suggested a strong correlation among the ratio of OS milestone rates at 12 months, ratios of OS milestone RMSTs at 12 and 24 months, and HR for OS. The results indicate that OS milestone rates and OS milestone RMSTs could be considered surrogate endpoints of OS in future first-line immunotherapy trials for ES-SCLC.

## Background

Small cell lung cancer (SCLC) is a life-threatening cancer, and the median overall survival (OS) for patients with extensive-stage SCLC (ES-SCLC) is only 8–10 months. Recently, immunotherapy has attracted increasing attention as a favorable treatment for SCLC. The combination of immune checkpoint inhibitors (ICIs) and chemotherapy, as the first-line treatment for ES-SCLC, has been reported to significantly improve OS compared with chemotherapy alone (1, 2). To date, four phase III and several phase II studies have been published regarding first-line immunotherapy in ES-SCLC. However, the appropriate surrogate endpoints of OS in first-line immunotherapy treatment for ES-SCLC remain largely unknown.

Data from patient- and trial-level studies have shown that PFS is strongly correlated with OS in first-line treatment of ES-SCLC and is a potential surrogate endpoint of OS (3, 4). Disease control rate (DCR) and duration of response (DOR) are strong predictors of OS in relapsed SCLC and are surrogate endpoints of relapsed SCLC (5). Immunotherapy has a unique response pattern, and its survival kinetics is different from those of chemotherapy. In studies comparing immunotherapy with chemotherapy, for example IMPOWER133 study, two survival curves often overlap or intersect for the first 6 months; the survival curves do not diverge until approximately 6 months of the study. Long-term survival is achieved only in some patients (the platform appears at the tail of the curve). Under these circumstances, the suitability of PFS, ORR, or DCR as surrogate endpoints for OS in first-line immunotherapy for ES-SCLC should be evaluated. Since the survival curve of immunotherapy no longer follows the assumption of constant proportional hazards, the median OS cannot interpret all the characteristics of the survival curve; hence, immunotherapy trials face challenges in statistical design. Researchers are proactively exploring indicators that can promptly and accurately assess the effect of immunotherapy on OS of patients with ES-SCLC.

Recently, milestone survival and restricted mean survival time (RMST) have been explored as potential surrogate endpoints in immunotherapy trials. Milestone survival analysis is a cross-sectional assessment of OS at a clinically significant prespecified time point (6), which can capture the delayed clinical effect of immunotherapy. RMST has also been defined as the area under the survival curve for a specified time window (7, 8); it is a mean value. In studies with RMST as the endpoint the difference in RMST between the experimental and control groups represents the absolute benefit of OS. Several studies have used RMST as an endpoint. For example, KEYNOTE-604 study (9) used RMST as an endpoint of exploration; KEYNOTE-598 study (10) used RMST at 24 months as an indicator for the interim analysis; in addition, Bpharm et al. used RMST to reinterpret the study results of the CheckMate057 study (11). These studies suggest that surrogate endpoints are worth exploring in clinical studies of immunotherapy. However, the endpoints of the ES-SCLC study were set mainly from the experience of cytotoxic drug. The purpose of our study on the surrogate endpoints of immunotherapy in ES-SCLC is to provide a more suitable method for evaluating the efficacy. It provides a reference for the future clinical study design and a more comprehensive and pertinent interpretation of the current results of immunotherapy studies.

Here, we investigated the significance of 12-month OS milestone rate and 12- and 24-month OS milestone RMSTs as surrogate endpoints of OS in first-line immunotherapy for ES-SCLC. We analyzed the existing data on immunotherapy in treatment-naïve ES-SCLC patients to determine optimal surrogate endpoints that can predict OS early, reduce costs, and accelerate the development of ICIs in SCLC.

## Methods

### Literature Search

**Figure 1:**
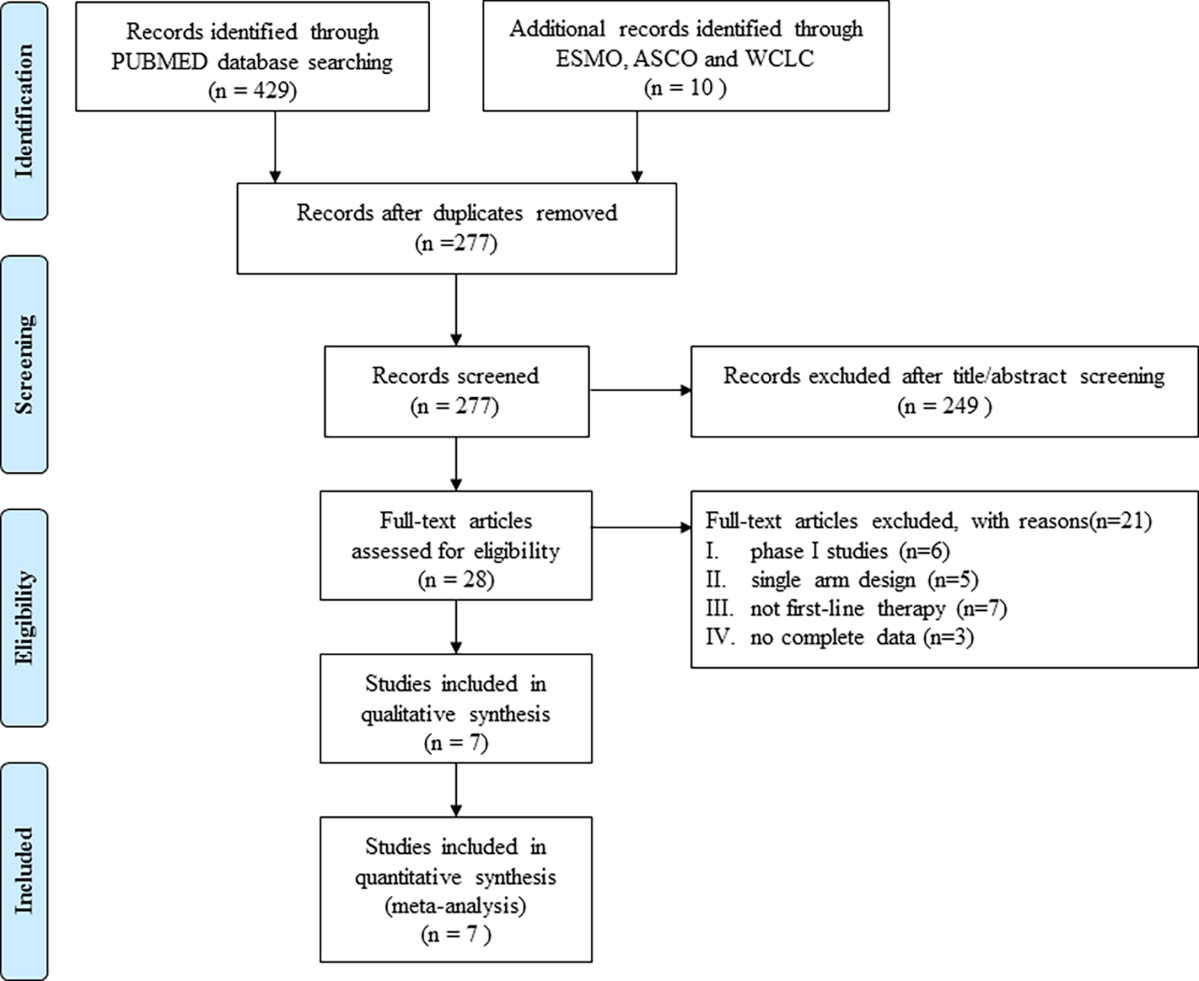
Flowchart of study inclusions and exclusions.

The randomized controlled phase II and III clinical trials of first-line immunotherapy for ES-SCLC, published between January 2013 and December 2020, were identified based on a systematic electronic search in PubMed. Abstracts from the European Society for Medical Oncology (ESMO), American Society of Clinical Oncology (ASCO), and World Conference on Lung Cancer (WCLC) reported since 2018 were also searched. The authors were independently involved in the literature search. Search terms included “small cell lung cancer OR SCLC”, “extensive disease”, “first-line treatment”, “PD-1/PD-L1”, “CTLA-4”, “pembrolizumab”, “nivolumab”, “atezolizumab”, “durvalumab”, “avelumab”, “ipilimumab” and “chemotherapy”. Relevant references of eligible clinical trials were also manually searched. A detailed flow diagram is presented in **Figure 1**.

### Data Collection

Two researchers (LS and ZS) separately extracted and cross-checked the data. Where there was a difference in opinion on any of the extracted data, consensus was reached by involving a third researcher who evaluated the same data and made the final decision. We extracted the following information from the included literature: name and phase of study, number of patients, experimental arm(s) regimen, control arm regimen, primary endpoint, and system for classification. The milestone rates were calculated using the Kaplan–Meier estimates. The model-independent values of RMST data were extracted from Kaplan–Meier curves using DigitizeIt Version 2.2 (www.digitizeit.xyz), and the area under the calculated curve was described according to a previously described method (12, 13).

### Statistical Analysis

Hazard ratios (HRs) were used to quantify the treatment effects on PFS and OS while RRs were used to quantify the treatment effects on ORR and DCR. The ratios of milestone RMST and OS milestone rates were used to quantify effects of the 12- or 24-month OS milestone RMST and 12-month OS milestone rates. Spearman rank correlation coefficients (rs) were calculated to evaluate the correlation between effects of treatment on surrogate endpoints and the HRs of OS. The correlation coefficient, r, ranged from −1 to 1 (an r value closer to 1 indicates a stronger correlation).

A weighted regression analysis based on the weighted least squares (WLS) method was performed on estimates of log-transformed treatment effect weighted by sample size of arms, and the determination coefficient R^2^ was calculated to reflect the strength of the association between treatment effects on the surrogate endpoints and HRs of OS. Data were analyzed using the R software (version 3.4.3; https://cran.r-project.org/bin/windows/base/old/3.4.3/). All tests were two-sided, and P-values <0.05 were considered statistically significant.

## Results

### Trials Included in the Analysis

**Table 1** lists the basic information of the included studies. Seven trials (three randomized phase II and four randomized phase III trials), representing 3,009 patients, for a total of 16 analysis arms were included (**Figure 1**). Two trials used the three-arm design, while five used the two-arm parallel control design. The primary endpoints of three trials were PFS, while two focused on OS and two focused on OS and PFS. Five trials investigated programmed cell death ligand 1(PD-L1)/programmed cell death 1(PD-1) inhibitors, while two assessed cytotoxic T-cell lymphocyte antigen-4 (CTLA-4) inhibitors. PFS and OS were reported in seven, ORR in six, and DCR in five studies. The 12-month milestone OS rate and 12-month OS milestone RMST could be extracted from seven trials, and the 24-month OS milestone RMST from six trials.

**Table 1:** Basic information of the included studies.

Study	Phase	Experimental Arm(s)	Control Arm	Primary endpoints	No. of patients	System for classifying response	Study arms
KEYNOTE-604 (9)	III	Pembrolizumab + EP/EC	Placebo + EP/EC	PFS and OS	453	RECIST 1.1	2
IMpower133 (1, 14)	III	Atezolizumab + EC	Placebo + EC	PFS and OS	403	RECIST 1.1	2
EA5161 (15)	II	Nivolumab + EP/EC	EP/EC	PFS	145	RECIST 1.1	2
CASPIAN (2, 16)	III	A:Durvalumab + EP/EC	EP/EC	OS	805	RECIST 1.1	3
		B:Durvalumab + tremelimumab + EP/EC					
Reck2012 (17)	II	A: phased-ipilimumab + paclitaxel + carboplatin	Placebo + paclitaxel + carboplatin	irPFS	130	mWHO& irRC	3
		B: concurrent-ipilimumab + paclitaxel + carboplatin					
Reck2016 (18)	III	Ipilimumab + EP/EC	Placebo + EP/EC	OS	954	mWHO	2
EORTC (19)	II	Pembrolizumab + EP/EC	Placebo + EP/EC	PFS	119	NR	2

### Analysis

**Figure 2:**
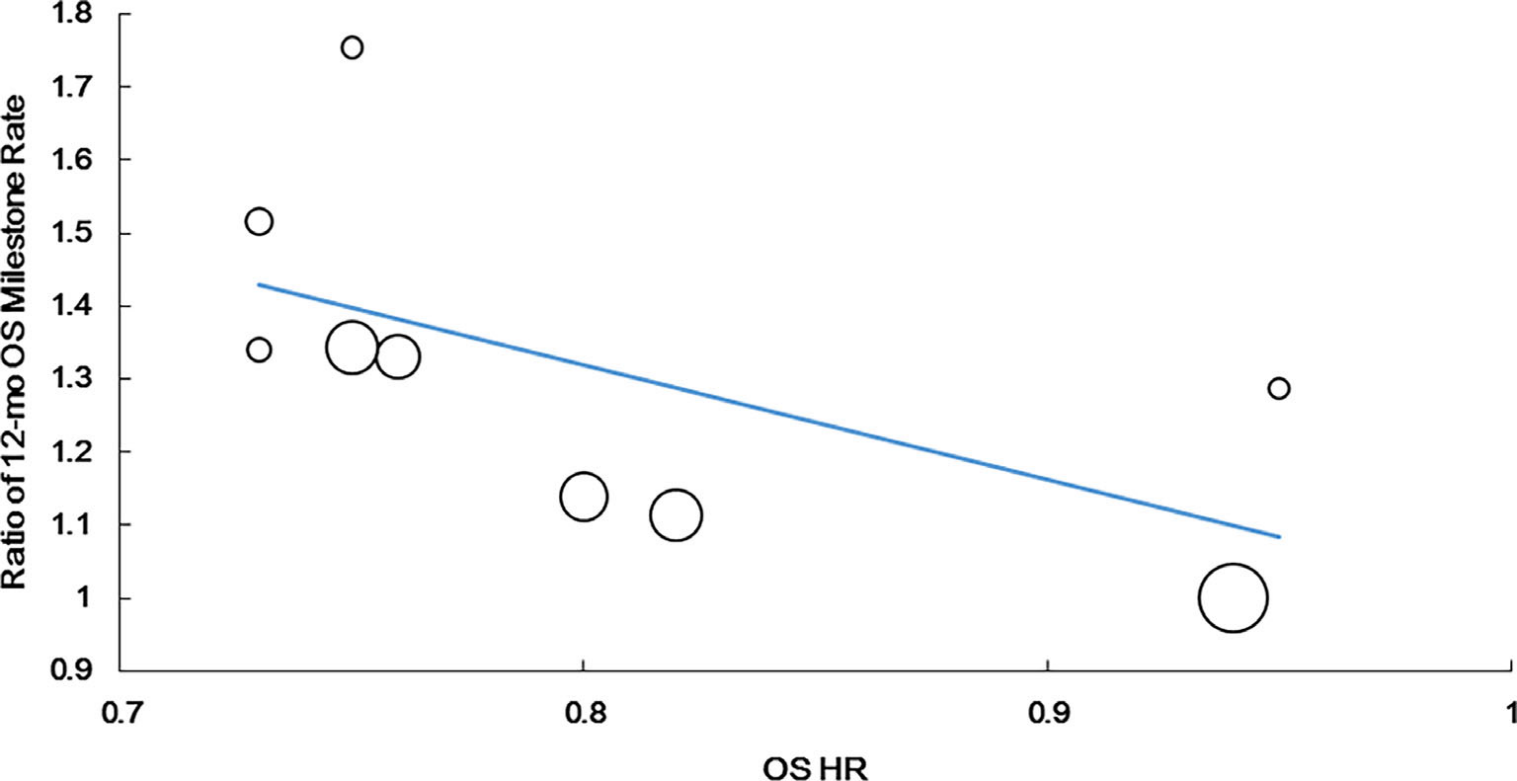
Correlation of treatment effects on the overall survival (OS) hazard ratio (HR) with the ratios of 12-month OS milestone rate.

A significantly strong positive correlation was identified between the 12-month OS milestone rate and HR for OS (r = −0.790, P = 0.011, n = 9). The weighted regression model was as follows: Log (HRos) = −0.099 − 0.567 × log (ratio of the 12-month OS milestone rate). The R^2^ value of the weighted regression line was 0.717 (P = 0.004), indicating that 71.7% of variability among the effects on OS could be explained by the ratio of the 12-month OS milestone rate (**Figure 2**).

Since KEYNOTE-604 (9) reported 12- and 24-month RMSTs of PFS and OS, we conducted sensitivity verification between the recalculated RMST and the reported data. The results showed that recalculated RMSTs were identical to data reported in the original articles (**Table S1**). Meanwhile, 12-month OS milestone rate, 12/24-month OS milestone RMSTs, HR of PFS, HR of OS for all included trials were shown in **Table S2**; estimated median OS and HR of OS and that compared with original reported data were listed in **Table S3**.

**Figure 3:**
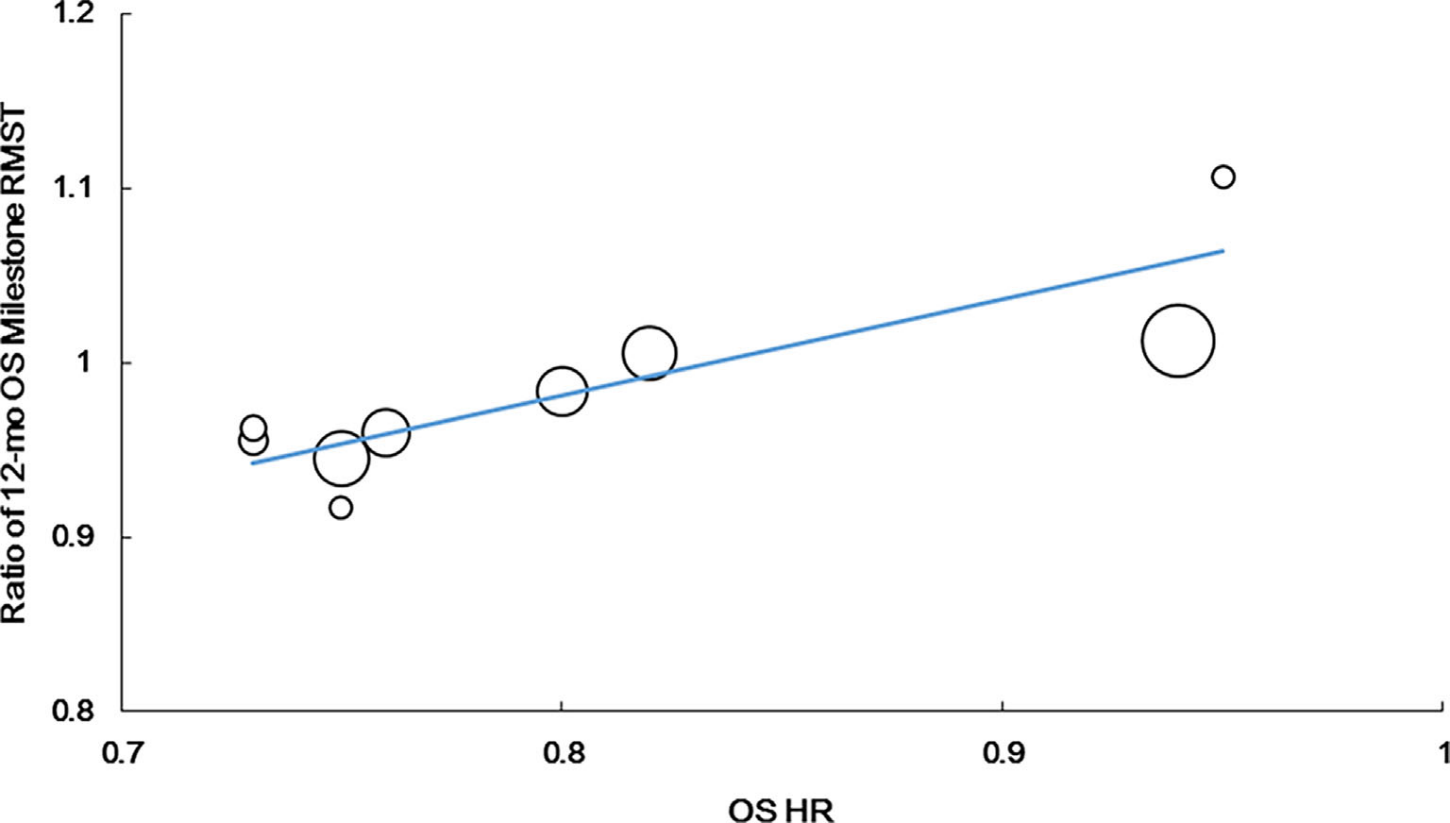
Correlation of treatment effects on the overall survival (OS) hazard ratio (HR) with the ratios of 12-month OS milestone RMST.

The ratio of the 12-month OS milestone RMST was strongly correlated with the OS HR (r = 0.798, P = 0.010, n = 9). The following regression formula was used: Log (HRos) = −0.160 + 2.337 × Log (ratio of the 12-month OS milestone RMST). R^2^ was 0.702 (P = 0.0048), suggesting that the ratio of the 12-month OS milestone RMST could explain 70.2% of HRos outcomes (**Figure 3**).

**Figure 4:**
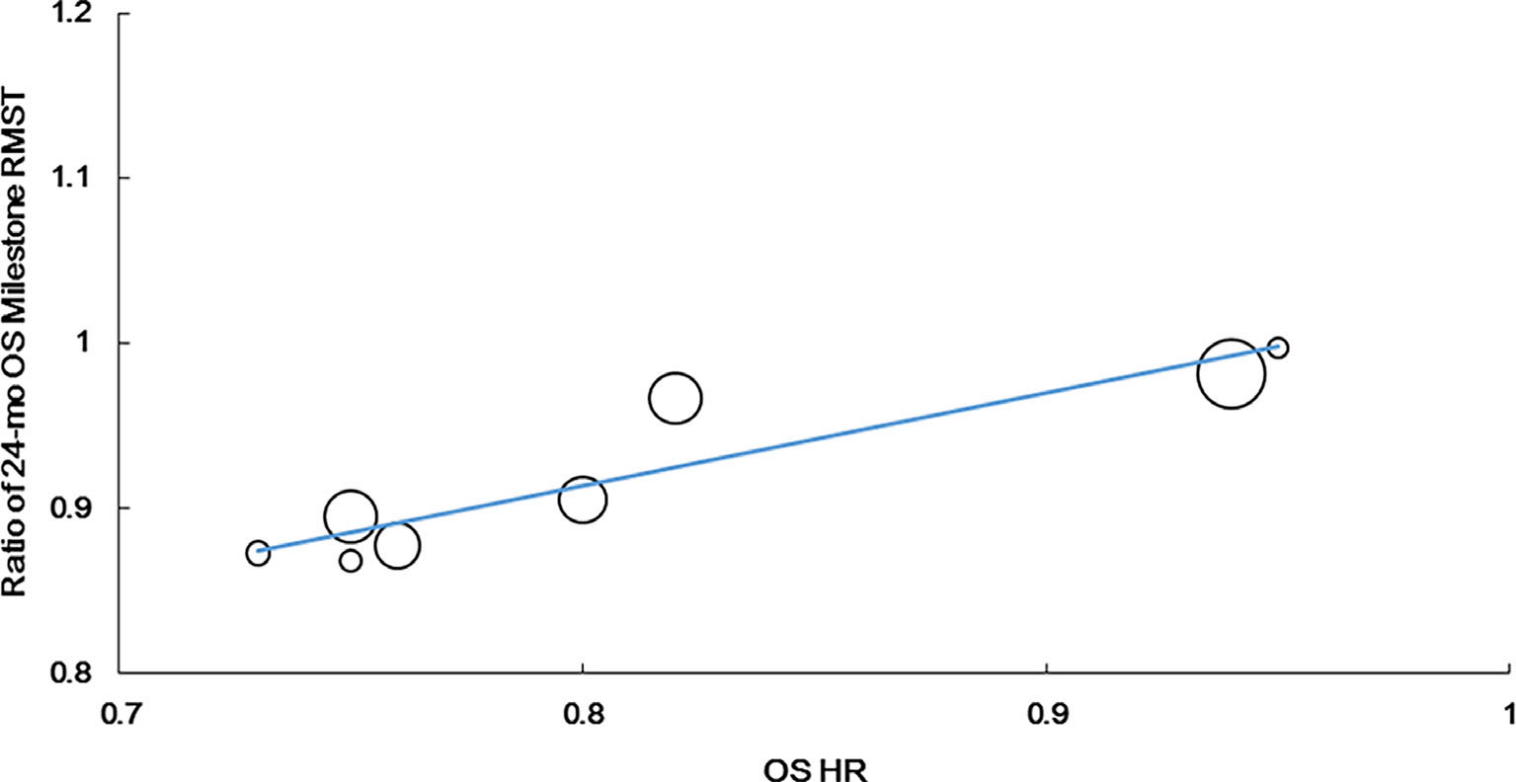
Correlation of treatment effects on the overall survival (OS) hazard ratio (HR) with the ratios of 24-month OS milestone RMST.

Additionally, we observed the strongest correlation between the 24-month OS milestone RMST and OS HR (r = 0.922, P = 0.001, n = 8). The equation for the resulting line was as follows: Log (HRos) = − 0.063 + 1.794 × Log (ratio of the 24-month OS milestone RMST). R^2^ was 0.825 (P = 0.002), suggesting that the ratio of the 24-month OS milestone RMST could explain 82.5% of HRos outcomes (**Figure 4**).

**Figure 5:**
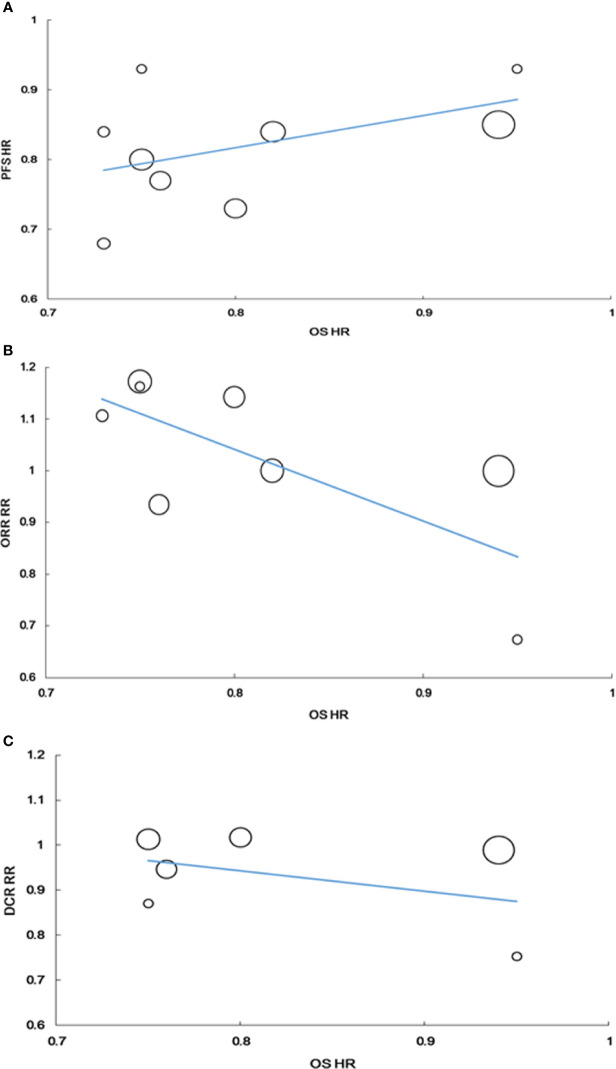
Correlation of treatment effects on the overall survival (OS) hazard ratio (HR) with the PFS HR **(A)**, with the RR of ORR **(B)**, with the RR of DCR **(C)**.

No correlation was found between the HR for PFS and HR for OS (r = 0.449, P = 0.225, n = 9). The weighted regression model was as follows: Log (HRos) = −0.033 + 0.758× Log (HR_PFS_); R^2^ was 0.315 (P = 0.116) (**Figure 5A**).

The RR for ORR tended to be correlated with the HR for OS, but the correlation was not statistically significant (r = −0.675, P = 0.066, n = 8). The weighted regression model was obtained using the following formula: Log (HRos) = −0.175 − 0.426 × Log (RR_ORR_); R^2^ was 0.233 (P = 0.226) (**Figure 5B**).

Similarly, no correlation was found between the RR for DCR and HR for OS. The correlation coefficient between RR for DCR and HR for OS was −0.232 (P = 0.658, n = 6). The weighted regression model was obtained using the following formula: Log (HRos) = −0.185 − 0.229 × log (RR_DCR_); R^2^ was 0.018 (P = 0.798) (**Figure 5C**).

## Discussion

To our best knowledge, this is a specific study to evaluate the trial-level surrogacy endpoints for OS, focusing on first-line immunotherapy for ES-SCLC. We found a strong correlation between OS HR and the ratio of OS milestone rates at 12 months or the ratios of RMST at 12 and 24 months. However, no correlation was observed between the HR of OS and PFS and the RR of ORR and DCR, which were unreliable surrogate endpoints of first-line immunotherapy for ES-SCLC.

A study by Chen et al., using 42 trials, evaluated the roles of PFS, ORR, and DCR as surrogate endpoints for OS in first-line therapy for ES-SCLC (20). Although the HR of PFS could explain 72% of the HR outcomes of OS, only three immunotherapy trials were included in this study. In addition, it was found that all three immunotherapy trials were below the weighted regression line. Consistent to the results of our study, the analysis of the correlation between the HR of OS and RR of ORR and DCR, suggested that the OS of immunotherapy cannot be accurately evaluated using ORR and DCR.

Although PFS was a potential surrogate endpoint for OS in ES-SCLC patients treated with chemotherapy, PFS was frequently inconsistent with OS in patients from the trials of first-line immunotherapy for ES-SCLC. In the CASPIAN study (2), compared to chemotherapy alone, durvalumab plus chemotherapy did not prolong PFS; however, it led to a statistically significant improvement in OS. In our study, no correlation was found between the HR for PFS and OS, respectively. This finding indicates that in the era of immunotherapy, PFS is no longer an ideal surrogate endpoint for OS as a first-line immunotherapy for ES-SCLC.

In a milestone survival analysis, Blumenthal et al. (21) observed a strong correlation between the 12-month OS milestone rate and OS HR in NSCLC immunotherapy studies. In our study, there was a very strong correlation between the HR for OS and the ratio of 12-month OS milestone rate of first-line immunotherapy for ES-SCLC. The OS milestone rate can be used as a potential surrogate endpoint for OS. Both the IMpower133 study and CASPIAN study considered the estimated number of OS events as the interim analysis time point; interim analyses of the two studies were performed at a median follow-up of 13.9 and 14.2 months, respectively, at approximately 60% maturity of OS (1, 2). However, the 12-month OS milestone rate analysis could predict the OS of first-line immunotherapy for ES-SCLC approximately 2 months in advance. Further studies are needed to determine whether 12 months is the ideal time point to perform the OS milestone rate analysis. Besides, the OS milestone rate is a cross-sectional analysis at a predetermined time point (22), which makes it difficult to summarize the survival curve in its entirety. RMST represents the distribution of any time event at a presetting and clinically meaningful time point (8), which can explain all survival information before the presetting time point. It is an absolute measure of survival time and can robustly interpret therapeutic efficacy. In our study, the ratios of OS milestone RMSTs at both 12 and 24 months were strongly correlated with HR for OS, particularly that of the OS milestone RMST at 24 months. In the KEYNOTE-604 study (9), although PFS of the interim analysis was inconsistent with OS in the final analysis, the 12-month PFS and 24-month OS RMSTs were favorable for combined treatment with pembrolizumab and chemotherapy. This can explain the divergent results and suggests that the OS milestone RMST could more accurately predict OS. Further investigations of the OS milestone RMST as a surrogate endpoint of first-line immunotherapy trials for ES-SCLC in the future are needed.

### Limitations

Our study has several limitations. First, we did not acquire detailed individual patient data; we only evaluated data at the trial-level. Patient-level data may provide more reliable data support for the issue of surrogate endpoints as first-line immunotherapy for SCLC. Second, although our study included all first-line immunotherapy trials for ES-SCLC, the included studies were heterogeneous, comprising phase III and II studies, evaluation criteria of Response Evaluation Criteria in Solid Tumors (RECIST) version 1.1, modified World Health Organization (WHO) criteria, and immune-related response criteria (irRC). Third, our study found that OS milestone rate and OS milestone RMST were better associated with OS, both of which require a presetting time point for analysis. However, determining the ideal time point is challenging. If the effect of immunotherapy is assessed too early, it may not be sufficiently significant. Moreover, the curve of OS still overlaps at 6 months, as seen from several phase III studies of first-line immunotherapy for ES-SCLC. So, we calculated RMSTs at 12 months and 24 months. We found that RMSTs at 12 months and 24 months had a strong correlation with OS, respectively (R^2^ = 0.702 and R^2^ = 0.825). The correlation was statistically stronger at 24 months. In addition, the curves of OS of the three phase III studies approached the plateau about 24 months. Since only three phase 3 studies have been published, more data are needed to confirm whether RMST at 24 months is the most appropriate. Fourth, these indicators for predicting OS are statistically calculated which is not intuitive and convenient for clinicians to use. We suggest that there will be more intuitive and objective evaluation indicators in the future with the presentation of more clinical research data of SCLC immunotherapy and deeper exploration of the survival dynamics of immunotherapy. Finally, data included in our study were limited, as immunotherapy in ES-SCLC is still in its infancy. Moreover, up to now the studies published have just about one year follow-up.

## Conclusions

In this study, the ratios of 12-month OS milestone rate and 12- and 24-month milestone RMSTs were found to be strongly correlated with the HR for OS. OS milestone rate and OS milestone RMST are promising surrogate endpoints of OS in first-line immunotherapy trials for ES-SCLC. OS milestone survival rate and OS milestone RMST could predict OS earlier and more accurately and are worth considering as intermediate endpoints of first-line immunotherapy trials of ES-SCLC in the future.

## Data Availability Statement

The original contributions presented in the study are included in the article/**Supplementary Material**. Further inquiries can be directed to the corresponding author.

## Author Contributions

Conception and design: SZ, SL, and YiC. Administrative support:YiC. Provision of study materials or patients: SZ, SL and YaC. Collection and assembly of data: SZ, SL and PZ. Data analysis and interpretation: SL and SX. Manuscript writing: All authors. All authors contributed to the article and approved the submitted version.

## Conflict of Interest

The authors declare that the research was conducted in the absence of any commercial or financial relationships that could be construed as a potential conflict of interest.
