# Supplementary material for: Consideration of Surrogate Endpoints for Overall Survival Associated With First-Line Immunotherapy in Extensive-Stage Small Cell Lung Cancer
Source: Front Oncol. 2021 Jul 14;11:696010. doi: 10.3389/fonc.2021.696010 (PMC8316832; doi:10.3389/fonc.2021.696010)
Supplement: Supplementary file 1 [file Table_1.docx]

Table S1 Sensitivity verification between the recalculated RMST and the reported data

|  | **Original data of Keynote 604** | **Recalculate data** | **Absolute Difference** | **Relative Difference** |
| --- | --- | --- | --- | --- |
| 24-month OS milestone RMST for pembrolizumab plus EP | 12.77m | 12.85m | 0.08 | 0.63% |
| 24-month OS milestone RMST for placebo plus EP | 11.56m | 11.73m | 0.17 | 1.47% |
| 24-month PFS milestone RMST for pembrolizumab plus EP | 5.86m | 5.82m | -0.04 | -0.68% |
| 24-month PFS milestone RMS for placebo plus EP | 5.14m | 5.02m | -0.12 | -2.33% |
