# Supplementary material for: Consideration of Surrogate Endpoints for Overall Survival Associated With First-Line Immunotherapy in Extensive-Stage Small Cell Lung Cancer
Source: Front Oncol. 2021 Jul 14;11:696010. doi: 10.3389/fonc.2021.696010 (PMC8316832; doi:10.3389/fonc.2021.696010)
Supplement: Supplementary file 2 [file Table_2.docx]

Table S2 12-month OS milestone rate, 12/24-month OS milestone RMST, HR of PFS, HR of OS for all included trials

| **study** | **12-month OS milestone rate for treatment group** | **12-month OS milestone rate for control group** | **12-month OS milestone RMST for treatment group** | **12-month OS milestone RMST for control group** | **24-month OS milestone RMST for treatment group** | **24-month OS milestone RMST for control group** | **HR of PFS** | **HR of OS** |
| --- | --- | --- | --- | --- | --- | --- | --- | --- |
| KEYNOTE-604 | 45.1% | 39.6% | 9.23 | 9.08 | 12.77 | 11.56 | 0.73 | 0.8 |
| IMpower133 | 51.9% | 39.0% | 9.78 | 9.39 | 13.87 | 12.17 | 0.77 | 0.76 |
| EA5161 | 47.6% | 31.4% | 9.58 | 9.15 | NA | NA | 0.68 | 0.73 |
| CASPIAN-D | 52.8% | 39.3% | 9.89 | 9.35 | 13.61 | 12.18 | 0.8 | 0.75 |
| CASPIAN-D+T | 43.8% | 39.3% | 9.3 | 9.35 | 12.6 | 12.18 | 0.84 | 0.82 |
| Reck2012-phased-ipi | 50.0% | 28.5% | 9.81 | 9 | 12.08 | 10.49 | 0.93 | 0.75 |
| Reck2012-comcurrent-ipi | 36.7% | 28.5% | 8.13 | 9 | 10.52 | 10.49 | 0.93 | 0.95 |
| Reck2016-ipi | 40.0% | 40.0% | 9.62 | 9.74 | 12.26 | 12.03 | 0.85 | 0.94 |
| EORTC | 50.4% | 37.6% | 9.72 | 9.36 | 13.23 | 11.55 | 0.84 | 0.73 |
