# Supplementary material for: Consideration of Surrogate Endpoints for Overall Survival Associated With First-Line Immunotherapy in Extensive-Stage Small Cell Lung Cancer
Source: Front Oncol. 2021 Jul 14;11:696010. doi: 10.3389/fonc.2021.696010 (PMC8316832; doi:10.3389/fonc.2021.696010)
Supplement: Supplementary file 3 [file Table_3.docx]

Table S3 Estimated OS medians and HR of OS and that compared with original reported data

| **Study** | **Median OS in the treatment group (months)** | | | | **Median OS in the control group(months)** | | | | **OS HR** | | | |
| --- | --- | --- | --- | --- | --- | --- | --- | --- | --- | --- | --- | --- |
|  | **original** | **estimated** | **Absolute Difference** | **Relative Difference** | **original** | **estimated** | **Absolute Difference** | **Relative Difference** | **original** | **estimated** | **Absolute Difference** | **Relative Difference** |
| KEYNOTE-604 | 10.8 | 10.7 | -0.1 | -0.93% | 9.7 | 9.6 | -0.1 | -1.03% | 0.8 | 0.76 | -0.04 | -5.00% |
| IMpower133 | 12.3 | 12.3 | 0 | 0.00% | 10.3 | 10.4 | 0.1 | 0.97% | 0.76 | 0.79 | 0.03 | 3.95% |
| EA5161 | 11.3 | 11.3 | 0 | 0.00% | 9.3 | 9.4 | 0.1 | 1.08% | 0.73 | 0.77 | 0.04 | 5.48% |
| CASPIAN-D | 12.9 | 12.9 | 0 | 0.00% | 10.5 | 10.7 | 0.2 | 1.90% | 0.75 | 0.77 | 0.02 | 2.67% |
| CASPIAN-D+T | 10.4 | 10.2 | -0.2 | -1.92% | 10.5 | 10.7 | 0.2 | 1.90% | 0.82 | 0.82 | 0 | 0.00% |
| Reck2012-phased-ipi | 12.94 | 12.9 | -0.04 | -0.31% | 9.92 | 9.95 | 0.03 | 0.30% | 0.75 | 0.78 | 0.03 | 4.00% |
| Reck2012-comcurrent-ipi | 9.13 | 8.93 | -0.2 | -2.19% | 9.92 | 9.95 | 0.03 | 0.30% | 0.95 | 0.93 | -0.02 | -2.11% |
| Reck2016-ipi | 11.0 | 10.8 | -0.2 | -1.82% | 10.9 | 10.9 | 0 | 0.00% | 0.94 | 0.93 | -0.01 | -1.06% |
| EORTC | 12.3 | 12.2 | -0.1 | -0.81% | 10.4 | 10.4 | 0 | 0.00% | 0.73 | 0.72 | -0.01 | -1.37% |
